# A new look at the essence of the imaging photoplethysmography

**DOI:** 10.1038/srep10494

**Published:** 2015-05-21

**Authors:** Alexei A. Kamshilin, Ervin Nippolainen, Igor S. Sidorov, Petr V. Vasilev, Nikolai P. Erofeev, Natalia P. Podolian, Roman V. Romashko

**Affiliations:** 1 Department of Applied Physics, University of Eastern Finland, P.O. Box 1627, FI-70211, Kuopio, Finland; 2 Scientific-Technical Centre for Computational Optics, Photonics and Imaging, ITMO University, 197101 St. Petersburg, Russia; 3 Delfin Technologies Ltd., FI- 70210 Kuopio, Finland; 4 Faculty of Medicine, St. Petersburg State University, 199034 St. Petersburg, Russia; 5 School of Natural Sciences, Far-Eastern Federal University, 690950 Vladivostok, Russia; 6 Lab. of Precision Optical Measurement Techniques, Institute of Automation and Control Processes FEB RAS, 690041 Vladivostok, Russia

## Abstract

Photoplethysmography (PPG) is a noninvasive optical method accepted in the clinical use for measurements of arterial oxygen saturation. It is widely believed that the light intensity after interaction with the biological tissue *in vivo* is modulated at the heartbeat frequency mainly due to pulsatile variations of the light absorption caused by arterial blood-volume pulsations. Here we report experimental observations, which are not consistent with this model and demonstrate the importance of elastic deformations of the capillary bed in the formation of the PPG waveform. These results provide new insight on light interaction with live tissue. To explain the observations we propose a new model of PPG in which pulse oscillations of the arterial transmural pressure deform the connective-tissue components of the dermis resulting in periodical changes of both the light scattering and absorption. These local changes of the light-interaction parameters are detected as variations of the light intensity returned to a photosensitive camera. Therefore, arterial pulsations can be indirectly monitored even by using the light, which slightly penetrates into the biological tissue.

It is commonly accepted that photoplethysmography (PPG) is a non-invasive optical method which is used to detect blood volume changes in the microvascular bed of tissue^1^,^2^,^3^. PPG waveforms are used for calculation of arterial oxygen saturation and heart rate determination in pulse oximeters which are widely accepted for routine clinical care^4^,^5^. Conventional pulse oximeters operate at the red and infrared light, and use the transmission-mode photoplethysmography in which the light source and photodetector are situated at the opposite sides of tissue, e.g., across a fingertip or an earlobe^2^,^4^. Such geometry provides efficient interaction of the light with biological tissue, which includes the optical processes of multiple scattering, absorption, reflection, and transmission^6^. The key factors affecting the light intensity received by the photodetector are the blood volume, blood vessel wall movement, and the orientation of red blood cells^7^,^8^,^9^. The general consensus is that the PPG waveform originates from pulsatile variation in the tissue optical density caused by arterial pulsations which are the most significant^9^,^10^. Capillaries are noncompliant with minor variations of their size^11^.

Imaging photoplethysmography (iPPG) is a non-contact imaging method for mapping cardiac synchronous pulsations. iPPG usually operate in reflection mode where both the illuminating light source and photodetector are situated alongside each other. In the reflection-mode PPG, light interaction with the tissue depends on the penetration depth of the illuminating light. Attempts to develop imaging pulse oximeter were reported^12^,^13^ by using the red and infrared light which penetrates into the tissue for several millimetres, e.g., 2.5 mm at the wavelength of 810 nm^14^. However, several researchers from different groups reported observation of a large-amplitude time varying (or alternating current, AC) modulation of the PPG waveform under green light illumination (wavelength of 510–560 nm) in the reflection-mode photoplethysmography^15^,^16^,^17^,^18^,^19^,^20^,^21^,^22^. This observation is difficult to understand in the frames of the existing physiological model of PPG because the penetration depth of the green light is less than 1 mm^6^,^14^, which makes the probability of light interaction with pulsatile arteries being rather low due to their deeper subcutaneous position^23^.

In our recent study of the reflection mode iPPG at the wavelength of 525 nm^24^, we observed a phenomenon which even stronger contradicts with conventional assumption that the PPG waveform is directly caused by pulsatile variation of the blood volume in arteries. This is the observation of the PPG waveform, which is inverted in the time scale in respect to the typical sawtooth one with the faster transition from the end-diastole phase. Such inverted waveforms were observed in adjacent areas of the wrist and/or palm for all 17 studied subjects^24^. No reasonable explanation of this phenomenon has been proposed.

In this report, we present new experimental data, which demonstrate the importance of elastic deformations of the capillary bed in the formation of the PPG waveform. Considering the package of experimental observations, we propose a new model of light interaction with biological tissue *in-vivo* in which pulse oscillations of arterial transmural pressure mechanically deform the connective-tissue components of the dermis resulting in periodical changes of both the light absorption and scattering coefficient. We detect these local changes of the light parameters as modulation of local light intensity returned to the photosensitive matrix. Therefore, we can indirectly monitor arterial pulsations even by using the light, which slightly penetrates into the biological tissue. The proposed model qualitatively explains the experimental observations.

## Results

### Mapping the areas with maximal PPG amplitude

The measurements were carried out with 58 healthy subjects. Mapping the PPG signal amplitude in the palm and wrist areas was done by using an iPPG system with lock-in pixel amplification referred to as Blood Pulsation Imaging (BPI), which was developed in our group^20^,^25^. Before measurement the subject placed his palm on the sponge support. Taking a comfort position the subject was asked to avoid hand movements during 30 seconds of video recording. We used the green light (wavelength of 525 nm) for illumination of the subject’s arm.

**Figure 1:**
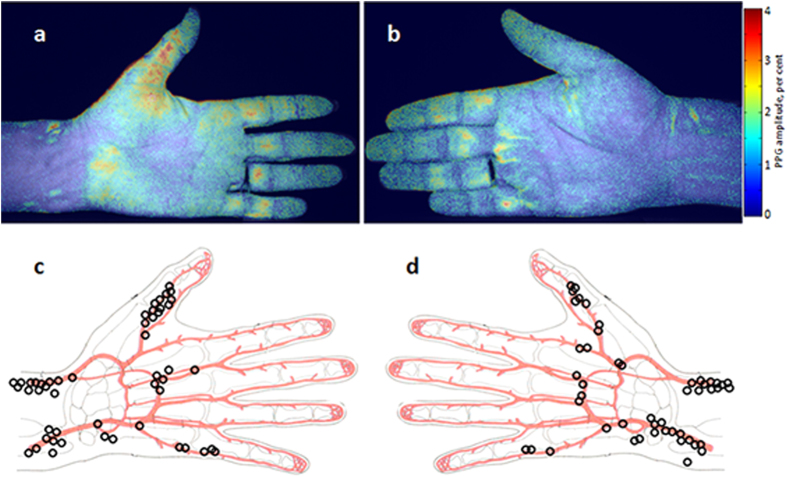
Mapping the PPG amplitude. Two examples of spatial distribution of the PPG amplitude (**a**) and (**b**) overlaid with raw camera images measured in two different subjects. Maps of the positions of the spots in which we observed the maximal PPG amplitude for the left hand (**c**) and for the right hand (**d**) as collected for all studied subjects: one black circle for one subject. The colour scale on the right shows the pulsation amplitude as the AC/DC ratio of the PPG waveform. The figure was drawn by A.K.

Two typical examples of the PPG-amplitude distribution are shown in Fig. 1a,b where the amplitude maps are overlaid with the initial camera image to provide an anatomic reference of the respective subject. The PPG-amplitude was calculated as an AC/DC ratio of the PPG waveform where AC and DC refer to the alternating (at the heartbeat frequency) and average (slowly varying) portions of the detected photo-signals, respectively. The amplitude is coded in Fig. 1a,b by pseudo colours so that the red colour corresponds to the higher amplitude. It is evident from Fig. 1a,b that the observed pulsations are unevenly distributed over the subjects’ arms. We found that these maps vary substantially from one subject to another. Evidently, for each subject we can find an area with the maximal PPG-amplitude (hot spot) and its position in the arm. We map the position of these spots in respect to the anatomical scheme of the palm with arteries separately for the left (Fig. 1c) and right (Fig. 1d) hands. In these maps, each hot spot belongs to one of the studied subjects. It is clearly seen that the most of hot spots are situated near either radial or ulnar artery. Nevertheless, there are subjects in whom the hottest spots are in the thumb or in the little finger. Anyway, the hottest spots are always observed in the vicinity of arteries. The amplitude of the blood pulsations in the hottest spots varies between 0.7% and 5.7%. Note that these values represent the AC/DC ratio, i.e. the amplitude of the alternating PPG-signal part modulated at the heartbeat frequency in relation to the magnitude of the slowly varying mean PPG signal. The mean amplitude in the hottest spot over all subjects was 2.2% (standard deviation, STD = 0.9%) for the left hand and 2.1% (STD = 1.0%) for the right hand.

### Counter phase PPG waveforms

**Figure 2:**
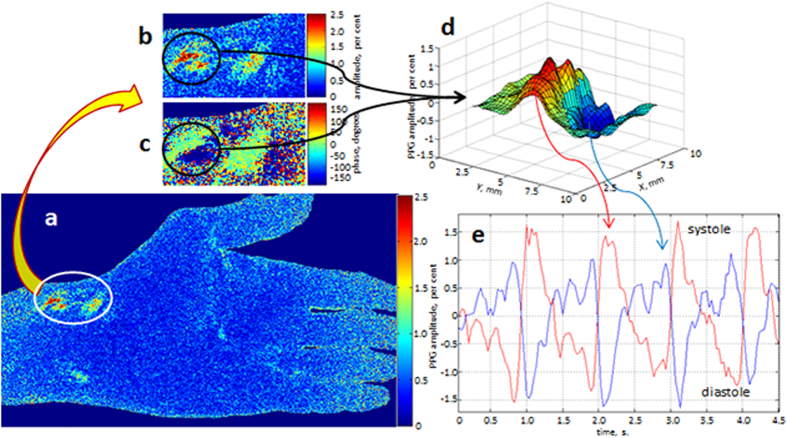
Counter-phase PPG waveforms. Map of the PPG amplitude (**a**) and the enlarged patch (**b**) of this amplitude map. A fragment from the map of blood pulsations phase (**c**) having the same size and position as the patch (**b**). Distribution of PPG pulsations (**d**) considering their relative phase in the area within the black circle shown in (**b**) and (**c**). PPG waveforms (**e**) calculated in ROIs of 3 × 3 pixels chosen in the points with extreme amplitude within the black circle. The colour scales alongside with pulsations maps are in degrees and in per cents for phase and amplitude PPG maps, respectively.

Experimental observation of the PPG waveforms pulsating in the counter phase was recently reported by our group^24^ taking an advantage of the BPI system to visualize the spatial distribution of the relative phase of blood pulsations. In the current study, we considerably extended the cohort of subjects and confirmed that all of them contain a pair or more adjacent areas in which the PPG waveforms oscillate with the same heart rate but in the counter phase. A typical example of such signals is shown in Fig. 2 where in Fig. 2a we show the map of PPG-amplitude for a subject whose maximal PPG amplitude is situated nearby the radial artery. An enlarged image patch of the area containing the maximal signal is shown in Fig. 2b. A fragment of the PPG phase distribution of the same area is shown in Fig. 2c. As seen in Fig. 2c, the phase difference between pulsations in the adjacent areas within the black circle is equal to 180 degrees. In Fig. 2d we show three dimensional (3D) plot of the pulsation-amplitude distribution considering the relative phase of pulsations for the area within the black circle in Fig. 2b,c. Note that two hot spots within this circle are separated by the distance of only 3.3 mm.

After determination the positions of the hot spots in the PPG-amplitude map in Fig. 2a, we place the centres of two regions of interest (ROI) in the points where two extremes of the amplitude were found. In these points we calculated the PPG signals by the spatial averaging the pixel values within the ROIs of the size 3 × 3 pixels, which corresponds to the area of 0.75 × 0.75 mm^2^ at the palm. These waveforms are shown in Fig. 2e. By convention used in the photoplethysmography literature^3^,^5^, all PPG waveforms in this paper are inverted in sign so that they positively correlate with varying transmural pressure. Therefore, the negative extremes of the PPG waveform correspond to the end-diastole phase. As one can see, the PPG signals in these adjacent areas indeed oscillate in the counter phase. It is worth noting that the red curve in Fig. 2e has a typical saw-tooth shape with the faster transition from the end-diastole phase, which is always observed in measurements of the arterial blood pressure waveforms^5^,^26^. In contrast, the blue curve is inverted in time and its shape clearly contradicts with behaviour of the arterial pressure. Erroneously, in our recent paper^24^ these counter-phase PPG waveforms were attributed with asynchronous blood supply to the adjacent areas because we tried to explain their origin in the frames of the commonly accepted model of PPG in which the signal is caused by pulsatile variation in the tissue optical density due to the blood-volume pulsations in arteries^10^. In the next Section we describe the new model of the PPG signal formation suggesting indirect impact of arterial blood pulsations on the light intensity modulation.

### Influence of the external local pressure

The experimental data presented in Figs. 1 and 2 were obtained in conditions when the subject’s palm was free of any contact. In the next experiment we studied the influence of the mechanical contact on the imaging PPG signals. To this end, a subject was asked to put his hand on the glass table while the illuminator (green light emitting diode, λ = 525 nm) and recording camera were settled under the table. To exclude direct reflections from the glass surfaces, we use linearly polarized light and a polarization filter installed before the camera with orthogonal orientation in respect to the polarization of the incident light. There were three steps of iPPG recordings. First recording was done when there was no contact of the skin with glass. In the second step, the contact of the palm with glass was provided by its own weight. The additional weight of 1.7 kg was applied to the hand in the third step. An additional pressure (its mean value) provided by this weight to the skin in the areas of the contact with the glass was estimated to be about 0.3 N/cm^2^.

**Figure 3:**
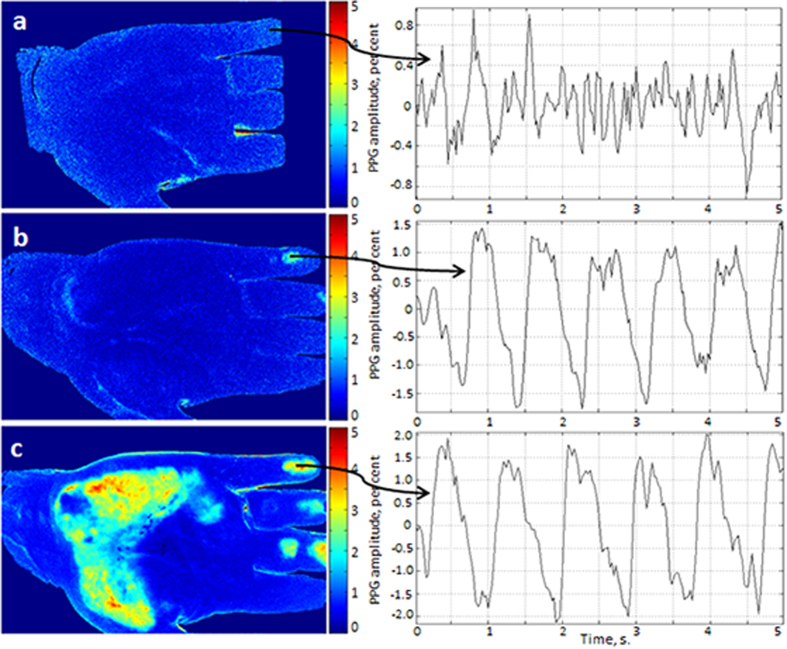
Spatial distribution of the PPG amplitude for different forces of the contact between the glass table and the skin of one of the subjects . (**a**)–noncontact iPPG; (**b**)–gentle contact; (**c**)–enforced contact by the distributed weight of 1.7 kg. Respective PPG waveforms are shown on the right being calculated in the ROIs of 3 × 3 pixels chosen in the same point of the little finger. The colour scales show the PPG amplitude as the AC/DC ratio in per cent.

The results of the experiment are shown in Fig. 3 where spatial distributions of the PPG-amplitude obtained during the steps 1, 2, and 3 are presented by images a, b, and c, respectively. As one can see, mechanical contact of the glass with the subject’s skin substantially increases the amplitude of the observed PPG signal. Moreover, by increasing the force of the contact (see Fig. 3c vs Fig. 3b), we increase both the amplitude of the PPG signal and the area with elevated PPG amplitude.

To estimate quantitatively the effect of the mechanical contact, we choose the ROI with the size of 3 × 3 pixels in the area of the maximal PPG amplitude obtained during the third step. It is at the last phalange of the little finger for the particular subject shown in Fig. 3. In the right side of Fig. 3 we show fragments of time-traces of the PPG signals calculated in the chosen ROIs for all three steps. The ROIs were situated in the same place of the finger for all three maps in Fig. 3. It is seen that in the case of absence of an external force applied to the skin, the PPG waveform has irregular, noise-like shape (see Fig. 3a). The mean PPG amplitude calculated as the AC/DC ratio of the signal averaged during 24 s is equal to 1.1% (peak-to-peak) in this case. One can hardly find quasi-periodical oscillations (which could be expected for arterial pulsations) in this case.

In contrast, even gentle contact with the glass considerably changes the PPG waveform which becomes more typical with a fast rising part after end-diastole phase (Fig. 3b). The mean amplitude of pulsations in this case is 2.96%, which is almost three folds higher than the previous. Video recording in the step 2 was carried out 2 minutes later after the step 1 while the recording in the step 3 was done after the delay of 1 minute. Additional weight applied in the step 3 to enhance the contact force increases the mean PPG amplitude up to 4.1%. These data clearly indicate that the PPG waveform is strongly affected by the external pressure applied to the subject’s skin.

## Discussion

The light penetration depth into the skin is usually defined as a path at which the light intensity is decayed to the level of 1/e^6^,^14^. For the used green light (525 nm) it was estimated to be between 0.4 and 0.9 mm^6^,^14^. The arteries are typically situated more than 3 mm below the epidermis in the wrist, and more deeper in the palm^23^. Considering these data and assuming applicability of the modified Beer-Lambert law to light propagation in biological tissue^27^ in combination with stronger light absorption by the erythrocytes^28^, one can estimate that the AC/DC ratio could not exceed 0.7%. In contrast, we found that the mean PPG amplitude in the hottest spots of all studied subjects is 2.1%, which is three folds higher than the above estimated value.

On the one hand, the effect of the contacting force on the PPG signal was reported previously^29^,^30^,^31^,^32^. However, these reports mainly concern the study of the contact PPG probe operating at deeper penetrating infrared light when it applied to the finger. It was found that the ratio of AC/DC of the PPG signal is just negligibly increases at small contacting forces (less than 0.4 N) with subsequent decrease at larger contacting forces^30^. The effect of the contact force was mainly attributed with change of the cross-section of arteries and veins^30^,^31^. Only mechanical properties of big vessels but not the finger tissue mechanical properties were considered in the previous model^31^. Moreover, no comparison has been made between contactless and contact photoplethysmography. Here we report about considerable increase of the AC-to-DC ratio in the case of low contact force as compared with contactless PPG signal. Note that the additional pressure to the skin provided by the contact force in our experiments was substantially smaller than in previous researches^29^,^30^,^31^,^32^.

On the other hand, several research groups in the field of optical coherence tomography have reported about increase of the light penetration depth into the tissue under its mechanical compression, referred to as mechanical optical clearing^33^,^34^,^35^. However, the penetration increase was observed under strong external pressure (>10 N/cm^2^) in conditions of the large pressure gradients^34^,^35^. Moreover, the clearing effect occurs with a delay of tens of seconds after beginning of the mechanical compression. In our experiment, the external pressure was smaller than 0.5 N/cm^2^ with very small pressure gradients. Therefore, the effect of optical clearing at compression is hardly achievable in our case.

Reported experimental findings (especially that one related to the counter phase PPG waveforms) cannot be explained by using commonly accepted model of photoplethysmography, which assumes that the light is modulated by the periodical blood volume changes caused by arterial pulsations. To explain the presented observations we propose a new model based on the well-known physiological postulate that the considerable pulse-pressure oscillations take place only in arteries^26^. These oscillations occur at the heartbeat frequency and provoke considerable changes of transmural pressure in arteries. In its turn, the transmural pressure mechanically deforms adjacent tissues^36^ which contain both blood and lymphatic capillaries. These deformations may include change of the orientation or structure of the connective tissue. Consequently, elastic deformations of the dermis will result in variations of the back-reflected light intensity due to local changes of the both scattering and the absorption coefficient of the light.

**Figure 4:**
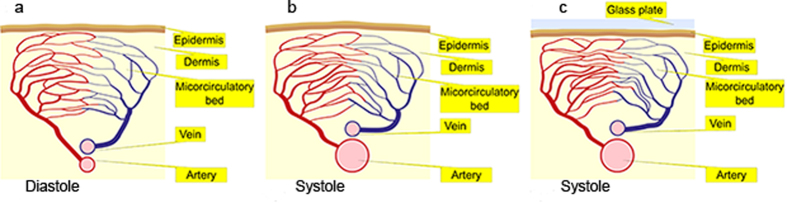
Simplified diagram of the new concept of the PPG signal formation. (**a**) A part of the artery, which is situated near the dermis, in the diastole phase; (**b**) the artery in the systole phase in the case of free skin surface; (**c**) the systole phase in the case of the skin contact with the glass. Note that the density of vessels in the microcirculatory bed is the lowest in (**a**) and the highest in (**c**). The figure was drawn by I.S.

The concept of the proposed model is depicted by a simplified diagram in Fig. 4. In the end-diastole phase (Fig. 4a), the arterial pressure is at its minimum, which suggests that the dermis is also under minimal stresses. We suppose that the light scattering coefficient of the dermis is minimal in this phase, as well. Therefore, the intensity of the back-reflected light is maximal in the end-diastole phase, which is well-established fact^13^,^18^. Then the fast increase of the arterial pressure during the systole provides the force which results in enlargement of the artery cross-section. If the subject’s skin is highly elastic (which is typical for young persons), the enlargement of the artery leads to reshaping of the skin surface with moderate compression of the dermis as shown in Fig. 4b. However, in the case of the mechanical contact of the skin with a glass (Fig. 4c), the dermis is compressed more significantly increasing the efficient density of capillaries. Both the absorption and scattering coefficient of the compressed tissue grows up resulting in diminishing of the reflected light intensity. This model is supported by *in vitro* measurements of optical properties of skin samples under compression^37^. It was shown in this study that both the absorption and reduced scattering coefficients of skin samples at the wavelength of 500 nm were increased several times after being compressed by the pressure of 0.1 N/cm^2^. Even though the thickness of the samples was two – three folds diminished under this pressure, the light transmittance through them was increased less than 10%^37^. Such insignificant optical clearing of the tissue could not be a reason of strong elevation of the PPG signal observed in our experiments after the contact with the glass plate.

Capillaries do not pulsate at the heart-beat rate^3^,^26^, which means that their filling with blood has minor variations during a cardiac cycle. However, the growing transmural pressure of the arteries during the systole compresses the connective tissues of the dermis in a local place, which results in the increasing density of capillaries. Consequently, the light propagating through this place will be stronger absorbed, which leads to the smaller intensity of the light returned to the photodetector. Therefore, the light intensity (PPG signal) is modulated due to changes of both scattering and absorption coefficients inversely proportional to the degree of the dermis compression. However, it is not clear now, which channel (absorption or scattering) prevails in modulation of the light intensity. Since the dermis compression is directly proportional to the transmural arterial pressure, the PPG signal follows the pressure after the inversion of its variable part. Note that in our graphs in Figs. 2 and 3 we change the sign of the alternating part of the PPG waveforms to achieve the positive correlation with the arterial pressure as it is often accepted in papers devoted to the photoplethysmography^3^,^5^,^38^. After such operation, the maximum and minimum of the PPG waveform are interchanged. Therefore, we are able to monitor pulsations of the arteries indirectly through elastic-mechanical interaction of deep arteries with the superficial dermis even by using the light, which cannot penetrate deeply into the tissue, e.g., green light.

In the contactless PPG experiment (Fig. 1), the pulsatile elastic-mechanical wave caused by the oscillating transmural pressure reaches the stratum corneum, and the amplitude of the stresses applied to the dermis is defined by the distance between the artery and the skin and by the mechanical properties (elasticity) of all tissues including the natural border, the stratum corneum. Therefore, we observe the maximal PPG amplitude in areas which are situated in the vicinity of arteries as it is confirmed by the experimental data shown in Fig. 1c,d. The position of the maximal PPG amplitude for a specific subject can be affected by the particular topology of his arteries and the status of his skin. For example, local defects of the dermis, such as old wounds could lead to another rate of tissue compression by the elastic pulsatile wave. It is worth noting that in the geometry of video recording in orthogonal polarizations used in our iPPG system, oscillations of the subject's skin do not affect the intensity of the reflected light because the reflection from the upper layer occurs in the same polarization state and thereafter filtered out by the second polarizer.

When the skin is led to a light contact with a solid barrier such as the glass window (Fig. 3b), the boundary conditions of the elastic-mechanical wave propagation in the dermis are changed. It results in the higher amplitude of the stresses applied to the dermis and consequently to the bigger amplitude of the PPG signal as one can see comparing Fig. 3a,b. Further increase of the total contact force applied to the arm leads to enlargement of the area in which the dermis is squeezed with the larger amplitude by the elastic-mechanical pulsatile wave. The increase of the amplitude of dermis squeezing is observed as high amplitudes of the PPG waveform, which is clearly seen in Fig. 3c.

Observed counter phase modulation of the PPG signal (see Fig. 2) can also be explained in the frames of the proposed model of light interaction with living tissue. The complex structure of the dermis may compose the conditions under which the mechanical compression of the dermis in one local place could lead to its expansion in the adjacent place. An example of such behaviour is an elastic rubber: application of the pressure to the centre of the peace of rubber leads to squeeze of its central part with the simultaneous expansion of the peripheral part. In this case, both the light scattering and light absorption in the centre will vary in the counter phase to these parameters in the periphery, which leads to the counter phase modulation of the light intensity in adjacent areas as one can clearly see in Fig. 2e.

Our observations show that it is elastic deformation of the dermis caused by transmural arterial pressure changes, which plays the key role in the formation of the PPG waveform when subject’s skin is illuminated by the light with small penetration length into the skin (at the wavelength of 510–560 nm). In the case of using deeper penetrating light (at the longer wavelength), the influence of the dermis compression is diminished because of more efficient interaction of the light with varying blood volume inside pulsating arteries. However, the rate of dermis-deformation influence is not spatially uniform since it depends on the local structure of the biological tissues and their structure at the moment of measurements. In some places, it can be considerable even for the infrared light which penetrates deeply into the biological tissue and efficiently interacts with arteries. The last decade has witnessed rapid growth in the literature pertaining to non-contact imaging photoplethysmography using the light with small penetration length^12^,^13^,^21^,^39^,^40^,^41^. We believe that the correct interpretation of non-contact iPPG results is impossible without taking into account the dermis deformation. As far as we know, modern multi-layers mathematical models of PPG^39^,^42^ do not consider dynamic variations of a layer thickness caused by arterial pulsations. Future development of an advanced mathematical model in which mechanical deformations of the biological tissues are taken into account would allow deeper understanding the nature of light interaction with living tissue and elaboration of new methods of vital sign monitoring.

## Methods

### Participants

The measurements were carried out with 58 healthy subjects. Age of the subjects was from 20 to 89 years. The group consisted of 17 women and 41 men. Persons with any neurologic, cardiovascular or skin diseases were excluded. All subjects gave their informed consent of participation in the experiment and in the publication of the results in the written form. This study was conducted in accordance with the ethical standards laid down in the 1964 Declaration of Helsinki. The study plan was approved by the Research Ethics Committee, Hospital District of Northern Savo.

### Measurement system

**Figure 5:**
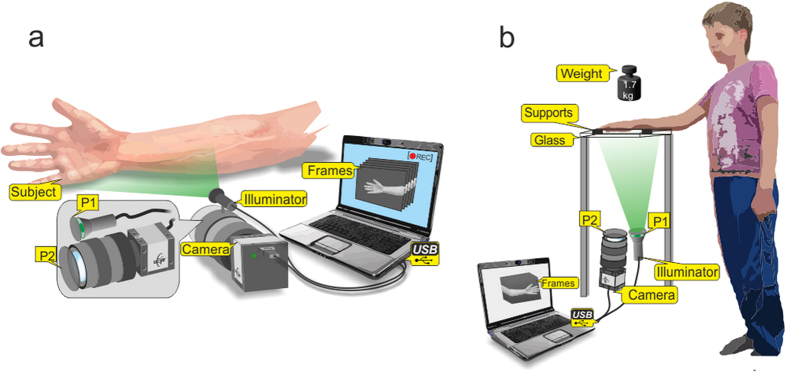
Schematic view of the experimental setups. (**a**) Mapping the position of the spots with maximal PPG amplitude, and (**b**) studying the influence of the mechanical contact on the PPG waveform. LEDs are light-emitting diodes; P1 and P2 are polarizers with orthogonally oriented transmission axes. The figure was drawn by E.N.

The experimental setup for recording the video frames of the subject was implemented in the reflection geometry as shown in Fig. 5. A custom made illuminator consisted of two light emitting diodes (LED) provided almost uniform illumination of the area of 25 cm × 40 cm at the distance of about 1 m. We used conventional LEDs H2A3-530 from Roithner Lasertechnik GmbH (Austria) operating at the wavelength of 525 nm. The optical power of each LED was 30 mW and their spectral bandwidth was 60 nm. The LEDs were powered from a battery of a laptop computer providing subject’s illumination with very stable light intensity. The illuminating light was linearly polarized by means of a polarizer P1 (Fig. 5) attached to the illuminator. The polarizer P2 was attached to the camera lens so that its transmission axis was oriented orthogonal to the polarization vector of the illuminating light. By using crossed polarizations we reject the direct reflections from the interface air/skin, air/glass, and glass/skin, which occur without change of the light-polarization state.

The light reflected from the illuminated area, which includes the palm and the part of the wrist, was captured by the digital black-and-white CMOS camera (8-bit model GigE uEye UI-5220SE of the Imaging Development Systems GmbH) with the attached lens (18–108 mm focal length, Canon, Japan). The direction of observation provided by the video systems made a small angle with the direction of illumination. The video frames with focused images of the illuminated area were downloaded to a personal computer via the universal serial bus (USB). The frame rate of video recording was 30 frames per second (fps) and the frame size was 752 × 480 pixels. Recording time was 24 sec. During video recording, the lens diaphragm and the camera exposure time were set so that the recorded frames did not contain saturated pixels (the maximal pixel value is 255 for 8-bit camera) but were as high as possible. All measurements were carried out in a laboratory maintained at a temperature of 22–24 °C and relative humidity of 30–50%. The intensity of ambient illumination in the laboratory was much lower than that provided by the LED illuminator.

### Experimental protocol

We used two different arrangements for measuring the spatial distribution of the PPG amplitude (Fig. 5). For searching the areas with the maximal PPG amplitude (the hottest spots), we asked subject to put his hand on a soft pad vertically to the table (see Fig. 5a) and keep it without movements during the video recording. To study the influence of the contact force applied to the skin, a subject was asked to put his palm horizontally over the glass table as shown in Fig. 5b. Three sequential video recordings were executed for each subject. First, we recorded 24-s video when the subject put his wrist and fingers on two soft black supports to provide no contact of the visible part of his palm with the glass. Second video of the same length (24 s) was recorded when the subject put his hand directly onto the glass with just a gentle contact. The final, third video was recorded in the same position of the hand as in the second step, but with an additional weight of 1.7 kg distributed over the hand.

### Mapping the PPG amplitude

The recorded video frames were processed offline by using custom software implemented in the MATLAB® platform. The image processing is based on the synchronous (lock-in) amplification of the set of recorded frames with a heartbeat frequency, which was described in details in our previous papers^20^,^24^. Briefly, a reference function required for synchronous detection of cardiovascular pulsations in the first approximation was formed from an arbitrary chosen ROI (10 × 10 pixels) of the recoded images. Using this function, we calculate the correlation matrix^24^ for one cardiac cycle, which allows us to map the amplitude of blood pulsations and to find areas with elevated amplitude. Then we choose one of these areas for formation of the final reference function with which the correlation matrices were calculated for nine sequential cardiac cycles. The amplitude maps shown in Fig. 1 through Fig. 3 are distributions which were averaged over 9 cardiac cycles.

These maps were used for searching the areas with maximal PPG amplitudes (hot spots). To this end, we use raw images for calculation of the PPG waveforms during the whole series of the recorded frames. These waveforms were calculated by the spatial averaging the pixel values within the ROIs of the size 3 × 3 pixels. Examples of the waveforms are shown in Fig. 2e and Fig. 3. After preliminary determination of the hot spot position in the PPG-amplitude map, we calculated 400 waveforms around the suspected centre of this spot by shifting the centre of each subsequent ROI for one pixel within the net of 20 × 20 pixels. For each PPG waveform we calculate the pulsation amplitude as the AC/DC ratio for every cardiac cycle. Then the mean amplitude was calculated within the whole recorded series. An example of the mean PPG-amplitude distribution in the net of 20 × 20 pixels is shown in Fig. 2d. The position of the spot with the maximal PPG amplitude for each subject was rescaled and marked in the common layout of palm arteries in Fig. 1c,d.

## Author Contributions

A.K. conceived the idea, supervised the research, and wrote the main text of the manuscript; E.N. and I.S. developed the instrument for the experiment; A.K. has prepared Fig. 1, 2, 3, I.S. has drawn Fig. 4, and E.N. has drawn Fig. 5; P.V. and N.E. discussed the results and commented on the manuscript; A.K., E.N., N.P., and R.R. performed the experiments and analysed the data. All the authors helped in writing the manuscript.

## Additional Information

**How to cite this article**: Kamshilin, A. A. *et al.* A new look at the essence of the imaging photoplethysmography. *Sci. Rep.* **5**, 10494; doi: 10.1038/srep10494 (2015).
